# The Integration of Proteome-Wide PTM Data with Protein Structural and Sequence Features Identifies Phosphorylations that Mediate 14-3-3 Interactions

**DOI:** 10.1016/j.jmb.2022.167890

**Published:** 2022-11-17

**Authors:** C. M. Egbert, L. R. Warr, K. L. Pennington, M. M. Thornton, A. J. Vaughan, S. W. Ashworth, M. J. Heaton, N. English, M. P. Torres, J. L. Andersen

**Affiliations:** 1- Fritz B. Burns Cancer Research Laboratory, Department of Chemistry and Biochemistry, Brigham Young University, Provo, UT, USA; 2- Department of Statistics, Brigham Young University, Provo, UT, USA; 3- Department of Biological and Environmental Sciences, Longwood University, Farmville, VA, USA; 4- Department of Computer Science, Brigham Young University, Provo, UT, USA; 5- Quantitative Bioscience Program, Georgia Institute of Technology, Atlanta, GA, USA; 6- School of Biological Sciences, Georgia Institute of Technology, Atlanta, GA, USA

**Keywords:** 14-3-3, phosphorylation, PTM, signaling, AKAP13, web tool

## Abstract

14-3-3s are abundant proteins that regulate essentially all aspects of cell biology, including cell cycle, motility, metabolism, and cell death. 14-3-3s work by docking to phosphorylated Ser/Thr residues on a large network of client proteins and modulating client protein function in a variety of ways. In recent years, aided by improvements in proteomics, the discovery of 14-3-3 client proteins has far outpaced our ability to understand the biological impact of individual 14-3-3 interactions. The rate-limiting step in this process is often the identification of the individual phospho-serines/threonines that mediate 14-3-3 binding, which are difficult to distinguish from other phospho-sites by sequence alone. Furthermore, trial-and-error molecular approaches to identify these phosphorylations are costly and can take months or years to identify even a single 14-3-3 docking site phosphorylation. To help overcome this challenge, we used machine learning to analyze predictive features of 14-3-3 binding sites. We found that accounting for intrinsic protein disorder and the unbiased mass spectrometry identification rate of a given phosphorylation significantly improves the identification of 14-3-3 docking site phosphorylations across the proteome. We incorporated these features, coupled with consensus sequence prediction, into a publicly available web app, called “*14*-*3-3 site-finder*”. We demonstrate the strength of this approach through its ability to identify 14-3-3 binding sites that do not conform to the loose consensus sequence of 14-3-3 docking phosphorylations, which we validate with 14-3-3 client proteins, including TNK1, CHEK1, MAPK7, and others. In addition, by using this approach, we identify a phosphorylation on A-kinase anchor protein-13 (AKAP13) at Ser2467 that dominantly controls its interaction with 14-3-3.

## Introduction

The human 14-3-3 family consists of seven structurally similar isoforms (β, γ, ε, η, σ, τ, and ζ) each expressed from a different gene. Although the number of isoforms differs among organisms, 14-3-3s are structurally and functionally conserved from plants to animals, reflecting their role in fundamental cellular processes, including cell cycle, apoptosis, and metabolism.^1–18^ 14-3-3s differ in the sequence of their flexible C-termini, which have not yet been structurally resolved and are still poorly understood, but may help explain how 14-3-3 isoforms are differentially regulated.^19–23^

Many textbook mechanisms of molecular biology have been discovered from the perspective of understanding how 14-3-3 docks to and controls client protein activity. These mechanisms include the control of cell cycle progression via 14-3-3-mediated regulation of the CDK1-regulating phosphatase CDC25,^4,24,25^ the control of cell death by 14-3-3 binding to the BH3-only protein BAD,^8,9^ the control of transcription factor access to DNA,^26–29^ and the regulation of various metabolic enzymes, kinases, and structural proteins. (reviewed in^14^) 14-3-3s exert their effect by docking to one or two phosphorylated Serines or Threonines on the client protein.^30–33^ These phosphorylations often occur in regions of high intrinsic disorder,^34–39^ perhaps reflecting the flexibility needed for a client protein to fit within the 14-3-3 phospho-binding groove. Because these client protein phosphorylations (often in pairs) can be added by different kinases, 14-3-3 has the ability to integrate multiple signaling inputs to produce one biological output, thereby acting as a biological logic gate.^40^ In turn, the effect of 14-3-3 binding on the client protein can vary. 14-3-3s can sequester proteins in different cellular locations (e.g., the nucleus or cytoplasm), contort the shape of the protein into active or inactive conformations, or affect the interaction of the client protein with other proteins.^14,30,40^

Although modern mass spectrometry has helped identify hundreds of 14-3-3 client proteins,^40–50^ our understanding of how 14-3-3 regulates most of these proteins lags far behind, leaving many undoubtedly important biological mechanisms undiscovered. In our group’s experience, the most critical bottleneck of this workflow is the identification of the phosphorylation sites that mediate 14-3-3 binding. This process is low throughput and resource-consuming, generally entailing the testing of numerous sites individually and in combination through mutagenesis and coimmunoprecipitation—a process that can take many months to years for a single client protein. Yet, without identifying these phospho-sites, it is essentially impossible to gain reliable insight into the effect of 14-3-3 on the client protein.

Historically, 14-3-3 docking sites have been described as following the general consensus sequence of RXXpS/TXP (mode I) or RXF/YXpSXP (mode II). However, as the list of 14-3-3 client proteins has grown, we have seen many 14-3-3 docking sites differ from this sequence. For example, although the +2 proline enhances 14-3-3 binding to phospho-peptides *in vitro*,^31^ it exists in less than half of experimentally validated 14-3-3 docking sites.^30,31,40,51^ In addition, the −3 Arginine may, at least in part, reflect the consensus sequence of the many AGC family kinases that phosphorylate 14-3-3 docking sites rather than a strict requirement for 14-3-3 docking, as several other amino acids (e.g., Serine, Leucine, and Histidine) are relatively common at the −3 position.^30,31,40,51^ Furthermore, client proteins often have numerous “red-herring” phosphorylation sites within sequences that resemble the 14-3-3 consensus docking site but have no measurable impact on 14-3-3 binding when tested in the lab. Nevertheless, mutational studies suggest that 14-3-3s do not interact promiscuously with off-target phosphorylated Serines and Threonines. Thus, protein features beyond the 6-amino acid consensus sequence must contribute to creating an optimal 14-3-3 docking site.^33,52^ This idea is supported by recent structures of 14-3-3 binding to different conformations of BRAF, which highlight a variety of contacts between 14-3-3 and BRAF outside of the 6-amino acid consensus phospho-site sequence.^53^

In this study, we analyze experimentally validated 14-3-3 docking site phosphorylations to identify protein features that distinguish docking phosphorylations from non-docking phosphorylations. We also mine publicly available phospho-proteome data to gain insight into the unbiased observation frequency of docking site phosphorylations compared to all other phosphorylations across the proteome. We find that the unbiased mass spectrometry observation frequency of a given phosphorylation can reliably predict many 14-3-3 docking site phosphorylations. In addition, the degree of predicted intrinsic disorder around the phosphorylation site can further improve these predictions. By combining these features with consensus site prediction, we improve our ability to predict 14-3-3 docking site phosphorylations that match poorly with the consensus sequence. Lastly, we incorporate all these features into a machine learning-based web-app, termed “14-3-3 site-finder”, which we use to identify a phosphorylation on the guanine nucleotide exchange factor AKAP13 that controls 14-3-3 binding.

## Materials and methods

### Analysis of PTM features from the SAPH-ire dataset.

Phosphorylation site features aggregated at the protein and protein family level for each 14-3-3 docking site were compared to 202,850 unique S/T phosphorylation sites contained within the SAPH-ire dataset (PMID 35696084), derived from phosphosite data curated from HPRD 9.0 (PMID: 18988627), PhosphoELM (PMID 21062810), PhosphoSitePlus (PMID 30445427), SySPTM (PMID 24705204), and dbPTM (PMID 26578568). Of the 170 unique 14-3-3 sites available, 115 were also found in and could be compared with the SAPH-ire dataset from 2019 (accessible via https://pearl.biosci.gatech.edu). Sites were classified as either matching a “14-3-3 interacting” site or “other” and statistically evaluated using the test for unequal variances in JMP 16.1 (SAS Inc.) and the Welch’s T test that is optimal for such dataset comparisons.

### Development of ML tool:

The training data set was a combination of previously identified 14-3-3 docking sites and extended from current literature. We webscraped the necessary data from IUPred2A and 14-3-3Pred. We generated a database of PTM observation frequencies from available data on PhosphoSitePlus through 2021. The tool was implemented using R with the “randomForest” library. All code is deposited here: https://github.com/lynsiewarr/14-3-3-Site-Finder.

### Maintenance of cell lines:

HEK293T cells were purchased from ATCC. Cells were cultured in DMEM supplemented with 10% FBS and at 37 °C with 5% CO_2_.

### Mutagenesis and cell transfection:

FLAG-TNK1 expression plasmid was purchased from GenScript. FLAG-AKAP13 was purchased from Addgene. Mutagenesis of constructs was done using Q5 Site-Directed Mutagenesis Kit (NEB), following the manufacturer’s protocol. Cells were plated the day prior to transfection. The transfection complex was prepared in a 1:4 DNA/PEI MAX ratio in DMEM, mixed and then incubated at room temperature for 20 minutes. After incubation, complexes were added to the cells and returned to the incubator. Media was aspirated and replaced after 6–8 hours.

### Immunoprecipitation and Western blot:

For TNK1 and AKAP13 immunoprecipitations, transfected HEK293T cells were washed and harvested in cold PBS. Cell pellets were lysed in either co-IP lysis buffer (10 mM HEPES KOH pH 7.5, 150 mM KCl, 0.01% IGEPAL) supplemented with protease and phosphatase inhibitors and rotated at 4 °C for 15 mins. Lysates were then homogenized by passaging through a 25G needle and centrifuged at 21000xg for 10 mins to clarify. Clarified lysates were collected for immunoprecipitation and western blot. For lysates were incubated with anti-DYKDDDDK G1 Affinity Resin (GenScript) or DYKDDDDK Fab-Trap^™^ Agarose (Chromotek) for 1 hour at 4 °C with rotation. The resins were washed with PBS three times. The coimmunoprecipitated proteins were eluted with SDS sample buffer and boiled at 95 °C for 5 mins. Lysates were resolved by SDS-PAGE and transferred to nitrocellulose membrane using iBlot2 Western Blotting System. Membranes were blocked with either 5% non-fat dry milk in PBS, intercept blocking buffer (Li-cor), or 5% phosphoblocker (Cell Biolabs) in PBST for 1 hour at room temperature. Primary antibodies against proteins of interests were diluted 1:1000 in blocking buffer and incubated with blot overnight at 4 °C. Proteins were visualized and quantified using infrared fluorescent secondary antibodies IRDye^®^ 800CW Goat anti-Rabbit (Li-cor, 92632211, 1:10000), IRDye^®^ 680RD Goat anti-Mouse (Li-cor, 926–68070, 1:10000) and Li-Cor Image Studio 5.0 software.

### Graphs:

PRISM GraphPad 9.0 was used for statistical analysis of data and generation of figure graphs.

## Results

### Identifying the predictive features of 14-3-3 docking site phosphorylations.

**Figure 1.**
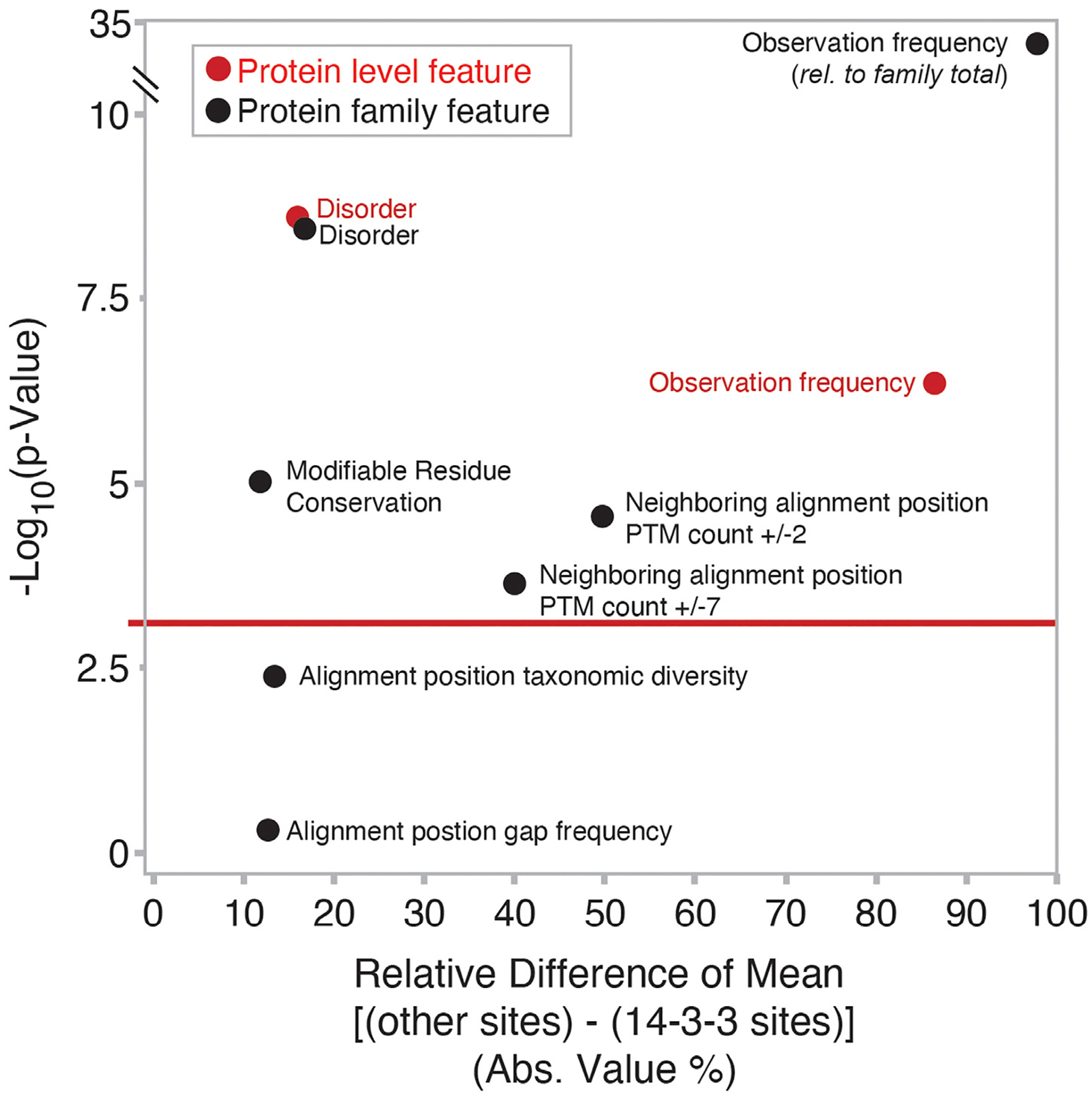
Statistical enrichment of features in 14-3-3 phosphorylated docking sites. Protein and protein family aggregated feature data were compared between 14-3-3 phosphorylation docking sites and 262,177 other phosphorylation sites. P-values were generated using Welch’s T test for unequal variances and plotted with respect to the relative difference in mean value for each population represented as a percentage (e.g. the absolute difference between the mean observation frequency values calculated for *other* versus *14*-*3-3* phosphosites at the protein level is 86.5%, corresponding to [|(4.4 – 32.6) / 32.6)| * 100]. Protein level features are those aggregated at the single protein level. Protein family features are those aggregated across the protein family of the 14-3-3 interacting protein. Significance threshold of P < 0.001 is indicated by the horizontal red line. *(see also* Table S1).

To begin to systematically extract features that could be used to identify 14-3-3 docking site phosphorylations, we compiled a dataset of known 14-3-3 docking site phosphorylations using previous lists from Mackintosh and colleagues^40,49^ and compared these with 202,850 unique S/T phosphorylations curated in 2019. These sites were organized further using *SAPH-ire*, a machine learning tool for predicting functional PTM sites that also provides a rich resource for PTM site features at both the protein and protein family level.^54,55^ From this we determined which protein and familial features were highly enriched in 14-3-3 phosphorylation docking sites compared to all other S/T phosphorylation sites. At the protein level we found that 14-3-3 docking phosphosites had significantly higher mean disorder and observation frequency compared to all other S/T phosphosites (Figure 1, Table S1). We observed a similar trend at the family level, but also found that 14-3-3 docking sites show a significantly higher than average modifiable residue conservation and higher than average frequency of neighboring modifications when looking +/−2 or +/−7 positions away from the phosphosite alignment position. In contrast, multiple sequence alignment features such as organism diversity contributing to the alignment and continuity of the aligned phosphosite (i.e. lack of gaps in the members contributing to the alignment position) were less/non significant descriptors. Based on this analysis and due to their independence from sequence alignment, we focused on disorder and observation frequency as strong distinguishing protein level features of 14-3-3 phosphosites.

**Figure 2.**
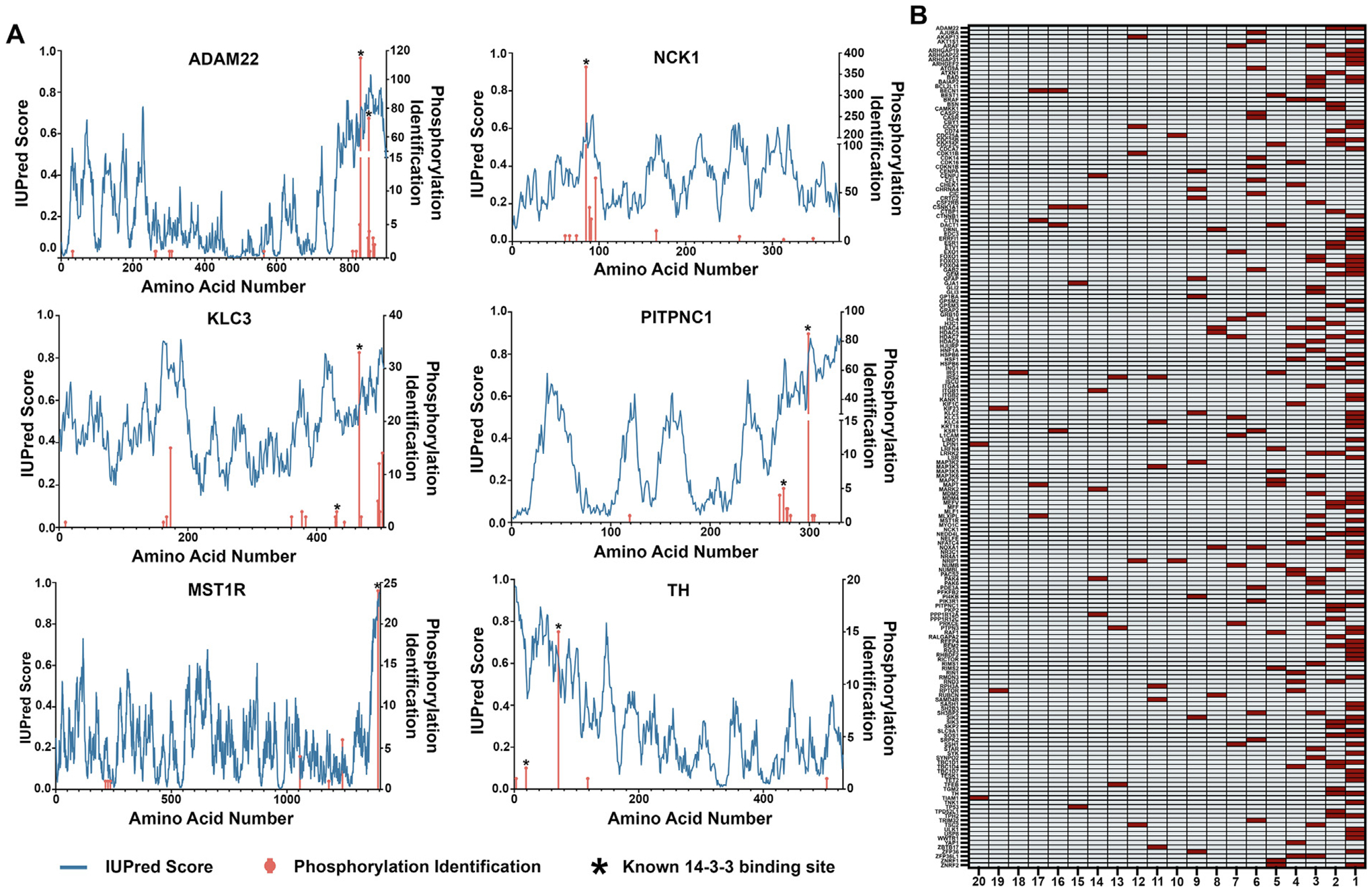
Evaluation of intrinsic disorder and unbiased MS observation frequency for 14-3-3 docking site phosphorylations. (A) Predicted disorder scores (IUPred) for each amino acid within the indicated protein are plotted (blue line and left axis) against the amino acid numbering. The number of identified Ser/Thr phosphorylation observations from Phosphositeplus.org are overlayed and shown as orange bars (right axis). Asterisk indicates known 14-3-3 docking phosphorylation(s). (B) Known 14-3-3 client protein Ser and Thr phosphorylations were ranked from most to least observed, based on unbiased and independent MS identifications logged at Phosphositeplus.org. The phosphorylated Ser/Thr site required for binding is indicated in red. The bottom axis represents the rank of the identified phosphorylation, with 1 being the most highly identified phosphorylation within the indicated protein. The top 20 most phosphorylated Ser/Thr sites (based on high throughput MS observations at phosphosite.org) are represented for each protein.

To help visualize relationships between intrinsic disorder, PTM observation frequency, and 14-3-3 docking site phosphorylations, we plotted IUPred disorder values for each amino acid of six 14-3-3 client proteins and superimposed these values over the MS observation frequency for each S/T phosphorylation sites. As shown in Figure 2(A), the 14-3-3 docking site phosphorylations typically fall within or just adjacent to regions of high predicted intrinsic disorder and are also among the most observed phosphorylations on the protein. To gain a broader view of the observation frequency of 14-3-3 docking site phosphorylations, we updated the dataset of 14-3-3 docking site phosphorylations from Mackintosh and colleagues^40,49^ with additional published studies that experimentally validate (e.g., by mutation) the docking site phosphorylations, giving a total of 327 phosphorylation sites across 234 proteins (Table S2). We randomly selected 14-3-3 client proteins from this data set and, using publicly available proteome-wide PTM data, we created a rank order of the phosphorylated Ser and Thr residues based on their MS observation frequency in unbiased high-throughput MS studies archived at PhosphoSitePlus.^56^ As shown in Figure 2(B), most 14-3-3 docking phosphorylations fall within the most frequently observed phosphorylations on a given client protein.

### Developing a machine learning model to predict 14-3-3 docking sites.

Next we asked whether integrating MS PTM observation frequency and/or the degree of intrinsic disorder around the PTM site with consensus sequence matching could improve predictions of 14-3-3 docking site phosphorylations. We took advantage of current prediction technology for 14-3-3 docking sites by using the web-based application developed by the Barton and MacKintosh groups called 14-3-3Pred (https://www.compbio.dundee.ac.uk/1433pred), which was shown by Madeira et al. to outperform other tools for 14-3-3 site prediction.^49,57,58^ 14-3-3Pred provides 3 different model outputs: an Artificial Neural Net (ANN), a Support Vector Machine (SVM), and a Position-Specific Scoring Matrix (PSSM). These outputs focus mainly on the amino acids within the binding region and their position relative to the pSer/Thr. Using these outputs, 14-3-3Pred defines sequence-matching cutoffs above which the score for a given site is classified as “high”, indicating a possible 14-3-3 docking site. Therefore, in total we used five different inputs for training our model: the ANN, SVM, and PSSM from 14-3-3Pred, a disorder score from IuPred2A, and a PTM score that we derived from PhosphoSitePlus.^56^ The PTM score reflects the frequency of unbiased LC-MS/MS-based observation for a given PTM. Importantly, because MS-based detection of PTMs is influenced by numerous factors (e.g., protein abundance, peptide solubility, etc.) and some proteins may be heavily phosphorylated while others are not, we generated a score to represent the PTM observation frequency normalized to the total number of phosphorylations on a given protein (equation below), providing a “PTM score’ that allows for fairer comparisons between different proteins.


$$\text{PTMscore}=\frac{\text{NumberofMS}-\text{basedobsevationsforagivenpSorpT}}{\text{TotalnumberofMS}-\text{basedobservationsforallphosphorylationsonthatprotein}}$$

**Figure 3.**
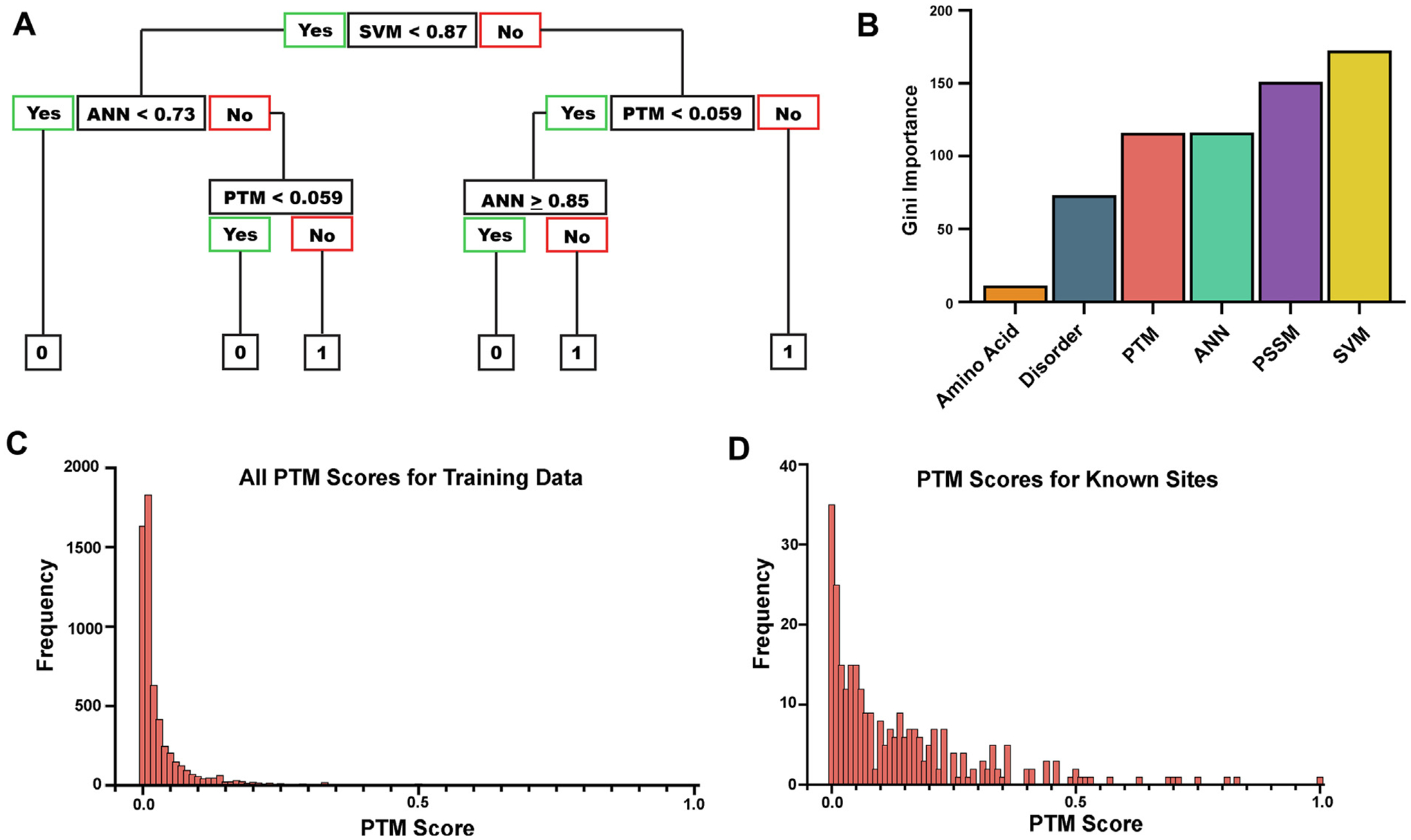
Random Forest modeling to evaluate the variables of intrinsic disorder and PTM score for predicting 14-3-3 docking site phosphorylations. (A) An example of a simple random forest decision tree. (B) The Gini importance calculations from random forest modeling. The higher the Gini importance value the more important the variable is in the prediction of a potential site. (C) PTM scores for every potential binding site within the proteins from the training data set were calculated and then graphed as a representation of the frequency of occurrence of each PTM value. (D) Using the data from C, only the PTM scores for known phosphorylated Ser/Thr sites required for 14-3-3 binding were graphed as a representation as frequency of occurrence as in C.

To begin assembling these features into a tool, we looked for an approach that uses stacking, like 14-3-3Pred, but also lists potential 14-3-3 docking sites by rank order as opposed to using cutoffs above which numerous sites may be essentially equally ranked and hard to prioritize for wet lab research. To accomplish this, we used a random forest algorithm, as described by Breiman.^59^ An advantage to this approach is its ability to capture nuanced relationships between different inputs. In addition, it is relatively quick to train, provides information about the importance of the variables used, and is generally good at prediction in classification settings. The random forest algorithm simulates several hundred decision trees by dividing the observations based on their characteristics in different ways. An example decision tree from our random forest model is shown in Figure 3(A). Predictions are made by running each new data point through all of the decision trees and selecting the outcome that most of the trees select as the best option.

We trained our algorithm using a variety of arrangements of the 5 different variables, giving our tool 3 different outputs, all of which form a list of predicted 14-3-3 docking sites from most likely to least likely. Within the app (https://rconnect.byu.edu/14-3-3-site-finder/), which we call “14-3-3 site-finder”, the first output is “Total Score”, which accounts for all 5 inputs (3 inputs from 14-3-3Pred primarily for sequence matching, an amino acid/protein disorder score from IUPred, and our PTM score). To visualize the relative contributions of the different inputs, we also generated random forest-modeled output columns for “PTM score and Disorder” and “14-3-3Pred scores” separately. We generated a fourth column, termed “Adapted 14-3-3Pred Score”, by using the specified cutoffs from 14-3-3Pred consensus scores, an average of the ANN, PSSM, and SVM scores, and adapting them into a rank order list for comparison to our other outputs within the tool.

To estimate the contribution of each variable in predicting 14-3-3 docking sites, we calculated the Gini Importance score, which establishes the degree to which removing a particular variable from the model would increase the node impurity on average, or the number of protein sites in each category that should have been classified into the other category for one tree. In short, the higher the Gini importance number, the more negative impact removing the variable has on the model’s predictive power. We found that SVM and PSSM are the most predictive variables by a slight margin, while the PTM score and disorder values follow closely behind, supporting their importance as 14-3-3 docking site features (Figure 3(B)). The contribution of disorder to 14-3-3 docking site prediction is not surprising given the well-established role of intrinsically disordered regions in mediating protein-protein interactions and with 14-3-3 client proteins specifically.^34–39^ However, we were surprised to see a strong contribution of the PTM score, which suggests that 14-3-3 docking site phosphorylations are often high stoichiometry PTMs. This may also reflect how 14-3-3-binding can shield phosphorylations from phosphatase activity, as in the case of CDC25, TET2, and others.^5,60–62^ Indeed, we see a clear upward shift in PTM scores of 14-3-3 docking site phosphorylations when compared to the PTM scores for all phospho-residues on proteins from our data set (Figure 3(C)–(D)).

**Figure 4.**
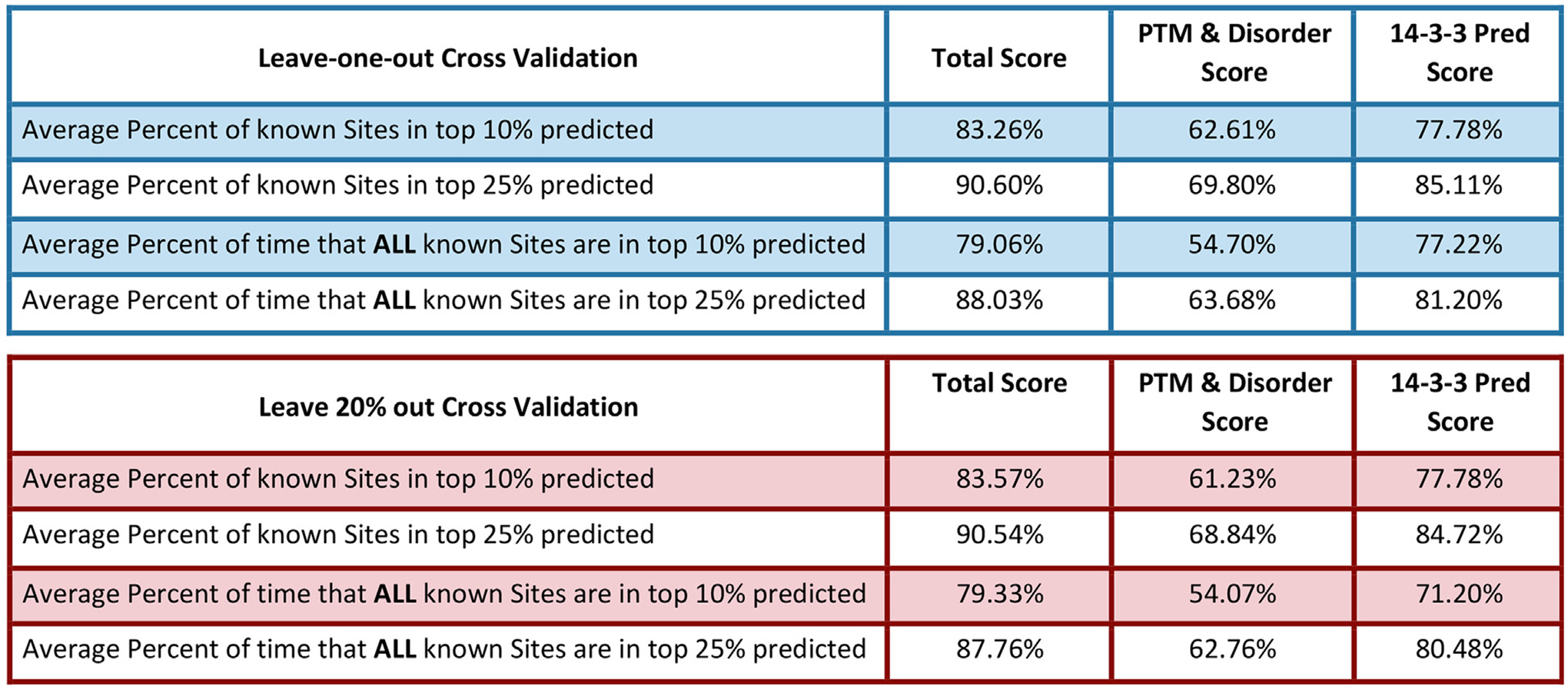
Validation of the 14-3-3 site-finder tool to identify 14-3-3 docking site phosphorylations. Summary of cross validation studies comparing the frequency of correct prediction in the top 10% or 25% of sites for each output column from the tool. The top panel uses a method that leaves a single protein out, retrains the model without that protein, and then uses the tool to predict 14-3-3 binding sites. We then determined whether the known 14-3-3 binding site was predicted within the top 10 or top 25 percent of sites predicted. The bottom panel leaves out 20 percent of the proteins from the training set, retrains the tool without the data, and then has the tool predict the 20 percent that were left out, with the same readouts as the top panel.

To validate the predictive ability of the 14-3-3 site-finder tool, we first performed a leave-one-out approach, in which we removed one protein at a time from the training set, trained the tool with that set, then tested the tool on the site that was removed—repeated for each protein in the dataset. In parallel, we also randomly removed 20% of our training data set and trained the tool on the remailing 80%. Once trained, we tested the tool’s ability to predict the 20% of sites that had been removed and repeated this procedure 200 times. We then calculated the percentage of time that the known site appeared in the top 10% and 25% of sites identified for each protein. Figure 4 shows a comparison of the tool’s predictive power when all 5 variables were included, compared to including only the PTM and Disorder scores versus relying on sequence prediction alone. For example, the correct site fell within the top 10% of predicted sites 83% of the time when all 5 variables were included versus 78% of the time when only sequence prediction was used (Figure 4). When we extend the results to the top 25% of sites, the tool has a success rate of almost 91% with all 5 variables taken into account, versus approximately 85% when consensus sequence is used alone (Figure 4).

### Identifying 14-3-3 docking sites that are poor matches to the consensus docking sequence.

We were initially surprised to see that despite the strong predictive power of PTM and disorder scores on their own, their combination with sequence prediction provided a relatively modest improvement of the tool’s predictive power. However, we noted that many of the sites included in the training data set were identified based on consensus sequence prediction, which likely biases the tool (and its validation testing) toward sites that closely match the historical consensus sequence for 14-3-3 docking sites.^40,49^ However, it is increasingly appreciated that many 14-3-3 docking sites do not conform to this consensus sequence.^35,51,63–68^ Therefore, we asked whether the inclusion of PTM and disorder scores would improve predictions for these non-canonical 14-3-3 docking sites.

**Figure 5.**
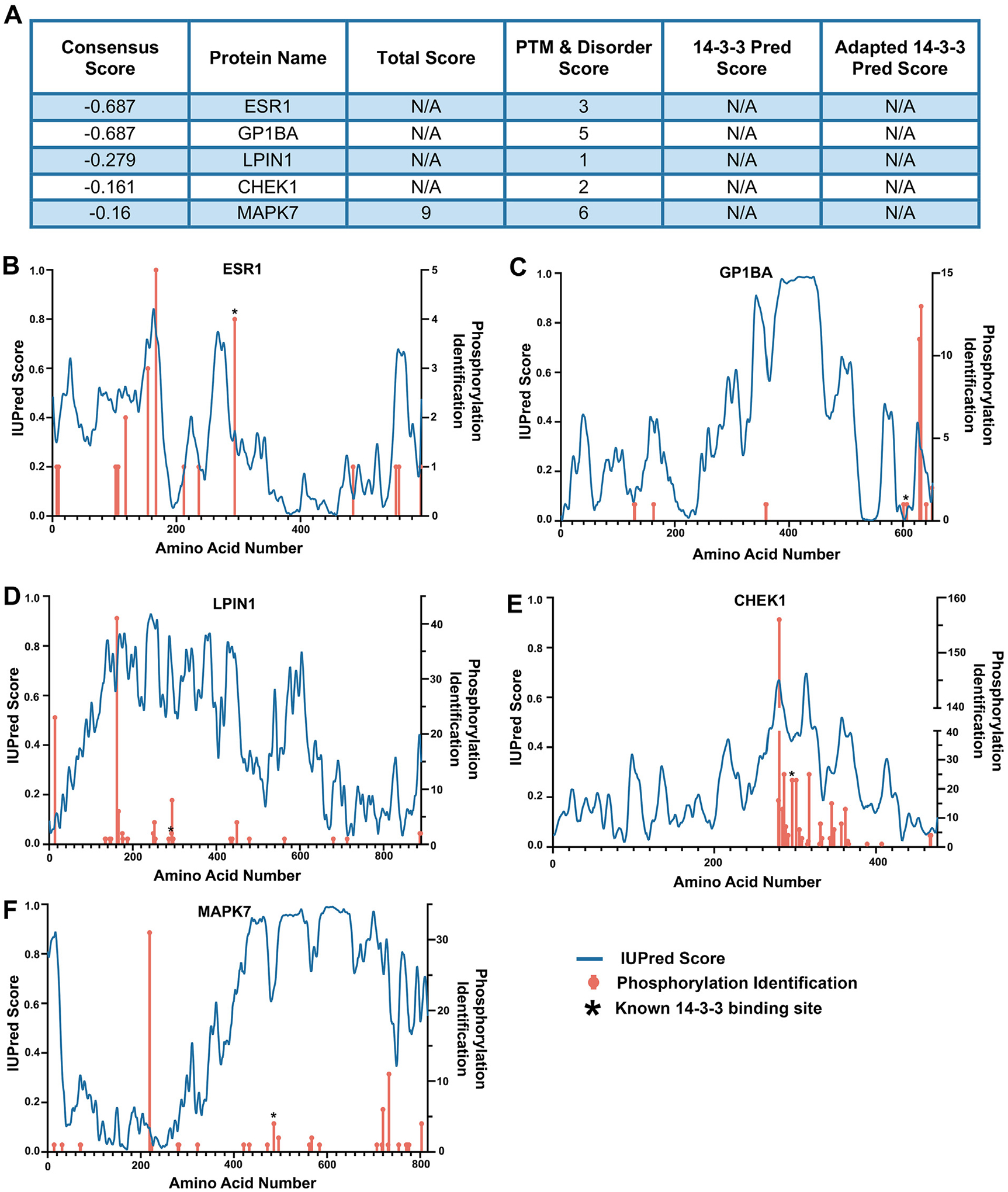
Identification of 14-3-3 docking sites that are poor matches to the consensus docking sequence. (A) Summary of the predicted ranks for the correct 14-3-3 docking sites of indicated proteins after they were removed from the tool and blindly predicted. (B)–(F) As shown in Figure 2(A), disorder scores from IUPred and Ser/Thr phosphorylation identifications from Phosphositeplus.org are graphed against the amino acid numbers for each indicated protein. Asterisk indicates known 14-3-3 docking site.

To identify non-canonical 14-3-3 docking sequences, we looked for proteins within our training data set (known 14-3-3 binding sites) that had the lowest consensus sequence scores, defined as any that fell below the 0.5 cutoff score of 14-3-3Pred. This gave us 68 14-3-3 docking site phosphorylations. Then we removed each of these sites from our training data set, retrained the tool, then allowed the tool to predict de novo the 14-3-3 docking sites on each protein in this set individually. We then set a generous cutoff, asking whether the 14-3-3 docking site was identified within at least the top 15 sites predicted by the tool. Interestingly, we found that for the small minority of proteins that lack a PTM score (i.e., the phosphorylation is not observed in aggregated MS studies), they consistently failed to rise above the top 15 cutoff, emphasizing the importance of the PTM score in instances where the 14-3-3 docking site phosphorylation is a poor match to consensus sequence. We then selected the lowest ranked (according to consensus score—i.e., a poor match to consensus sequence) 5 docking sites that also had PTM scores for further analysis. These were estrogen receptor-1 (ESR1) pS294, platelet glycoprotein Ib alpha chain (GP1BA) pS607, phosphatidate phosphatase LPIN1 (LPIN1) pS607, serine/threonine-protein kinase Chk1 (CHEK1) pS296, and Mitogen-activated protein kinase 7 (MAPK7) pS486. All five 14-3-3 docking sites on these proteins were predicted within the top 6 sites on each respective protein by the PTM & disorder score from our tool (data summarized in table in Figure 5(A)). Notably, the tool predicted pS607 of LPIN1 and pS296 of CHEK1 as the first- and second-ranked sites respectively. Figure 5(B)–(F) depicts plots of IUPred disorder values and PTM scores for each of the five selected 14-3-3 client proteins with the 14-3-3 docking site phosphorylation marked by asterisk.

As an alternative tool for comparison, we also trained the algorithm with the subset of 68 non-canonical docking sites (proteins below the 0.5 cutoff in consensus score) as well as a less stringent subset of non-canonical sites the fell below a 0.8 cutoff in consensus score. However, these tools failed to outperform the tool trained with the full set of client proteins in identifying non-canonical sites (Figure S1A), perhaps due to the small number of client proteins in the non-canonical training sets. Nevertheless, reducing the training sets to non-canonical sites raised the relative Gini importance scores for the PTM and Disorder variables, suggesting that in the absence of a good sequence match, the PTM and Disorder variables drive predictions to a greater degree (Figure S1B). Together, these data suggest that training the algorithm with the full current set of client proteins provides the best combination of variables for site predictions, while also underscoring the value of the PTM and Disorder scores in predicting 14-3-3 docking sites that do not conform well to the consensus sequence.

**Figure 6.**
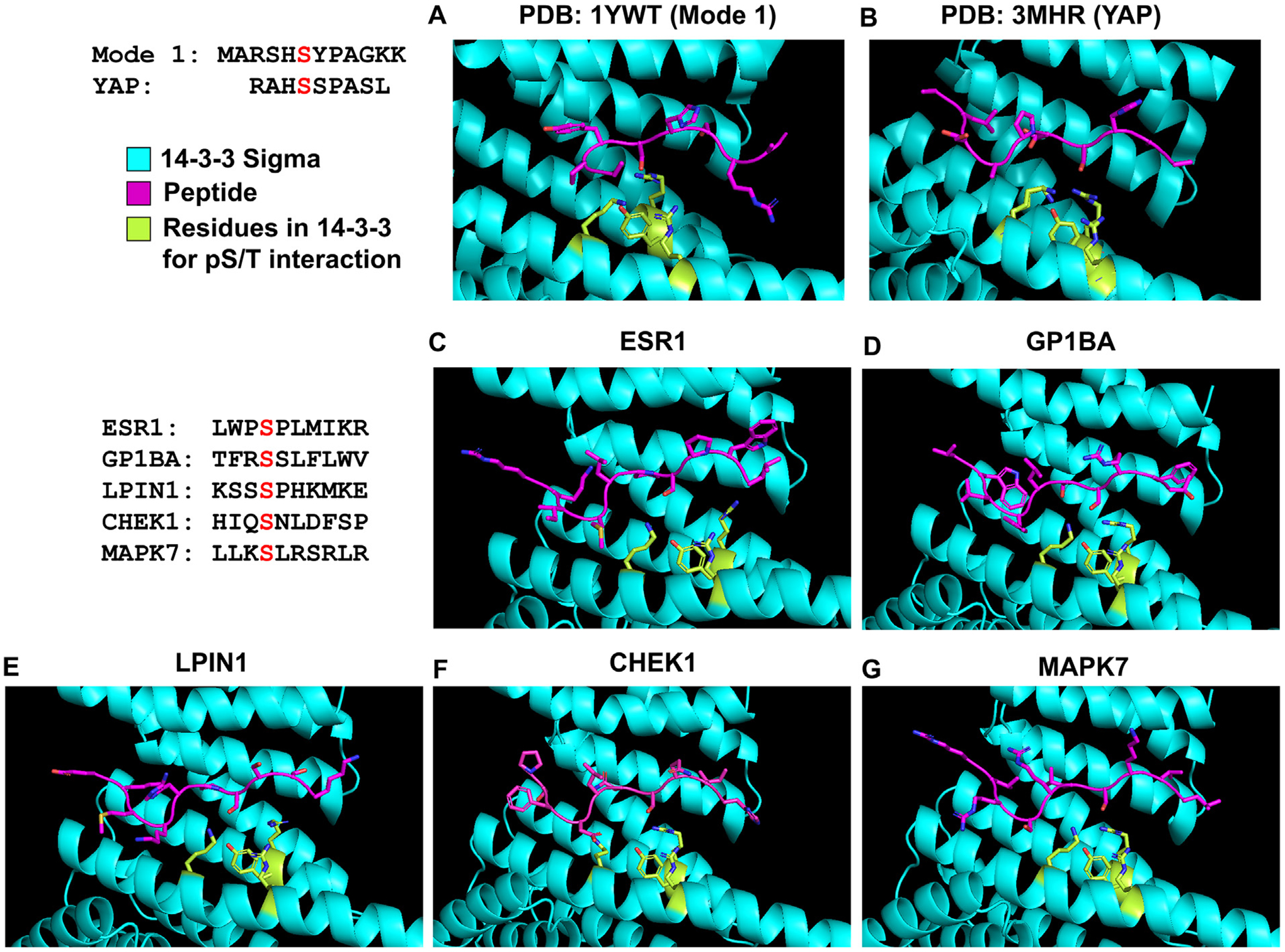
Structural modeling of non-canonical 14-3-3 docking sequences. (A) Structure of a mode 1 phosphopeptide with 14-3-3σ (PDB: 1YWT). (B) Structure of pYAP peptide bound to 14-3-3σ (PDB: 3MHR). (C)–(G) Structural modeling of the docking sites from the indicated proteins into the binding region of 14-3-3σ. 14-3-3σ is in cyan, peptides are in magenta, residues on 14-3-3σ important for interactions with phosphorylated Ser/Thr within binding peptide have sticks shown in green.

To examine these non-canonical 14-3-3 docking sites in more detail, we used structural modeling of 14-3-3 in complex with the docking site phosphorylation in question. As a homology model, we used the co-crystal structure (PDB: 1YWT) of 14-3-3σ in complex with a mode 1 phospho-peptide (MARSHSYPAGKK).^69^ In this structure, the phospho-peptide adopts a kinked/u-turn conformation at the +2 proline. We also used a co-crystal structure (PDB: 3MHR) of 14-3-3σ in complex with YAP pS127-peptide (RAHSSPASL)^64^ in which the phospho-peptide adopts a more extended/unkinked conformation (Figure 6(A) and (B)), serving as an alternate model to explain how a phospho-peptide may fit into the 14-3-3 binding groove. The phospho-peptide sequence for each non-canonical docking site was threaded onto the structure of the mode 1 peptide- or the YAP-14-3-3 models followed by side chain rotamer optimization and all-atom minimization of the peptide and then the entire structure in the Rosetta energy function using the academic standalone Foldit interface.^70^ To model our peptides, we applied constraints to mimic both polar interactions made by the phosphate group and hydrogen bonds observed in the 14-3-3σ:mode1/YAP peptide interface. Most of the docking sites fit within the binding pocket in the extended conformation (14-3-3σ: YAP) as shown in Figure 6(C)–(G). Key residues within the 14-3-3 binding groove that are known to interact with phospho-peptides,^31^ including Lys47, Arg56, Arg129, and Tyr130, are indicated in green. Our modeling suggests that these non-canonical sequences may adopt an extended/unkinked conformation within the 14-3-3 binding groove.

### TNK1 as a case study for 14-3-3 site-finder.

**Figure 7.**
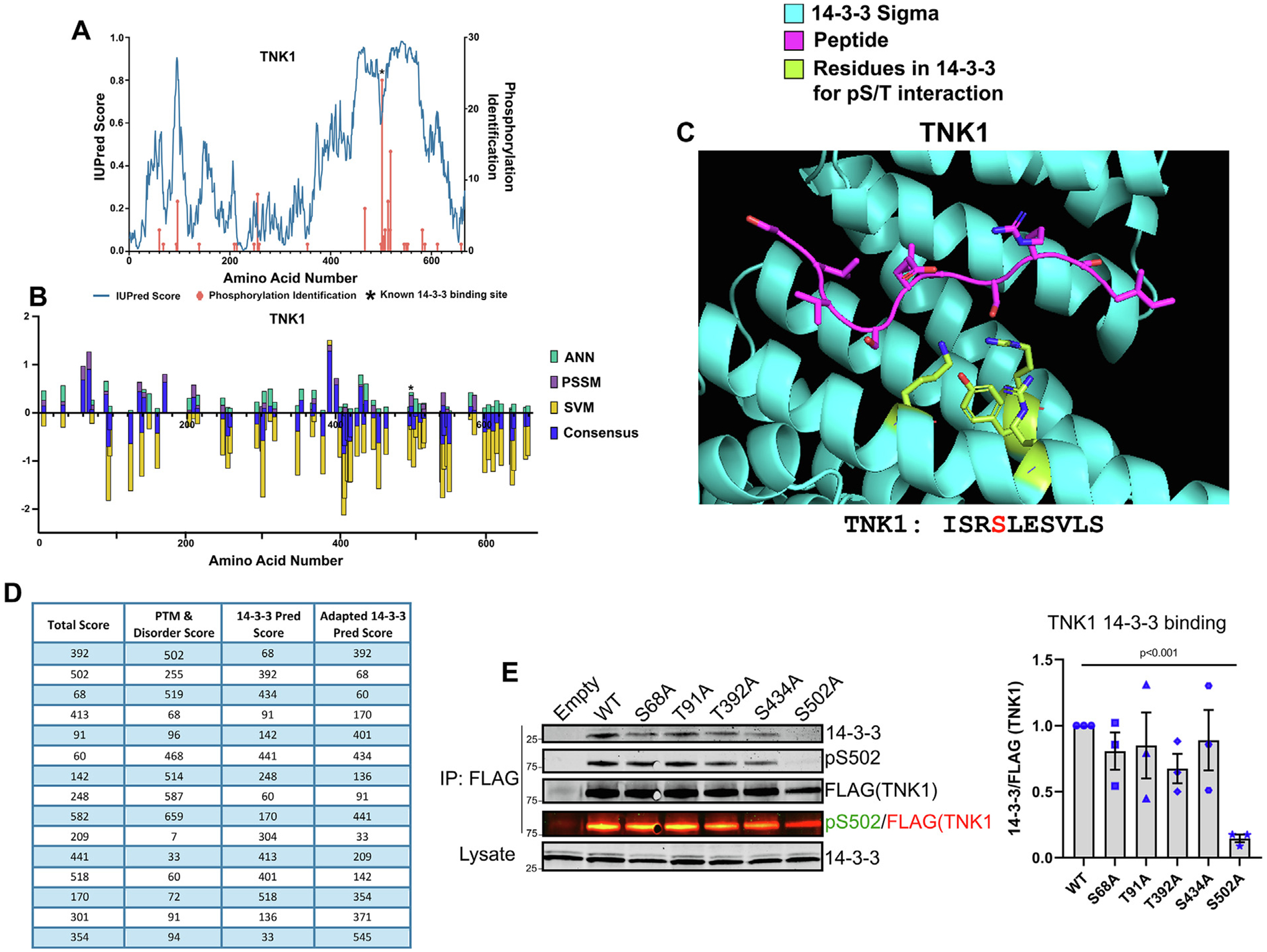
Applying the predictive features of 14-3-3-site-finder to TNK1 as a case study. (A) Disorder scores from IUPred and Ser/Thr phosphorylation identifications from phosphositeplus.org graphed against the amino acid numbers for TNK1, as seen in Figure 2&5. (B) Summary bar graph of ANN, PSSM, SVM, and consensus scores from 14-3-3Pred graphed against the amino acid numbers of TNK1. Asterisks in A-B indicate known 14-3-3 docking site. (C) TNK1 docking site modeled into the 14-3-3σ binding site as before (Figure 6). 14-3-3σ is in cyan, peptides are in magenta, residues on 14-3-3σ important for interactions with phosphorylated Ser/Thr have sticks shown in green. (D) Summary rank lists of the top 15 predicted docking sites for TNK1 by 14-3-3-site-finder for each output described. (E) Indicated mutated constructs of TNK1 were expressed in HEK293T cells followed by co-immunoprecipitation for 14-3-3 binding. Co-IP was immunoblotted for 14-3-3 and pS502 signal. Quantification of 3 biological replicates of 14-3-3 binding to FLAG (TNK1) on the right. Error bars indicate SEM for Students t-test.

In our previous work, we identified several 14-3-3 client proteins whose docking site phosphorylations were difficult to identify,^26,43,71^ which motivated the development of this tool. One of these 14-3-3 client proteins is the non-receptor tyrosine kinase TNK1, which we ultimately found to interact with 14-3-3 through a phosphorylation at S502 within the unusual docking sequence of ISRpSLES.^71^ As shown in Figure 7(A) and (B), pS502 is the most observed phosphorylation on TNK1 in unbiased MS studies and is within a region of high disorder, yet pS502 is not scored highly by sequence prediction. Also, modeling suggests the pS502 peptide adopts the same extended conformation within the 14-3-3 binding groove that we observed with other atypical docking site sequences (Figure 7(C)). In our earlier study on TNK1, we relied on a laborious protein truncation approach to identify the region of 14-3-3 binding, then used phospho-proteomics to narrow in on the docking site phosphorylation. Now in retrospect, as a case study, we questioned whether this tool could predict S502 as a docking site. Indeed, as shown in Figure 7(D), the Total Score, and PTM and Disorder score columns predict it as the second and first most likely site, respectively; whereas sequence-based approaches do not predict it within our top 15 site cutoff. We also questioned whether there may be a second docking site phosphorylation, which perhaps could be easily detected through consensus sequence-based approaches (14-3-3Pred). As shown in Figure 7(E), phospho-null mutations at the top 4 sites predicted by sequence failed to disrupt 14-3-3 binding to TNK1, while mutation of S502 completely abrogated binding.

### Applying the tool to identify a novel 14-3-3 docking site phosphorylation.

**Figure 8.**
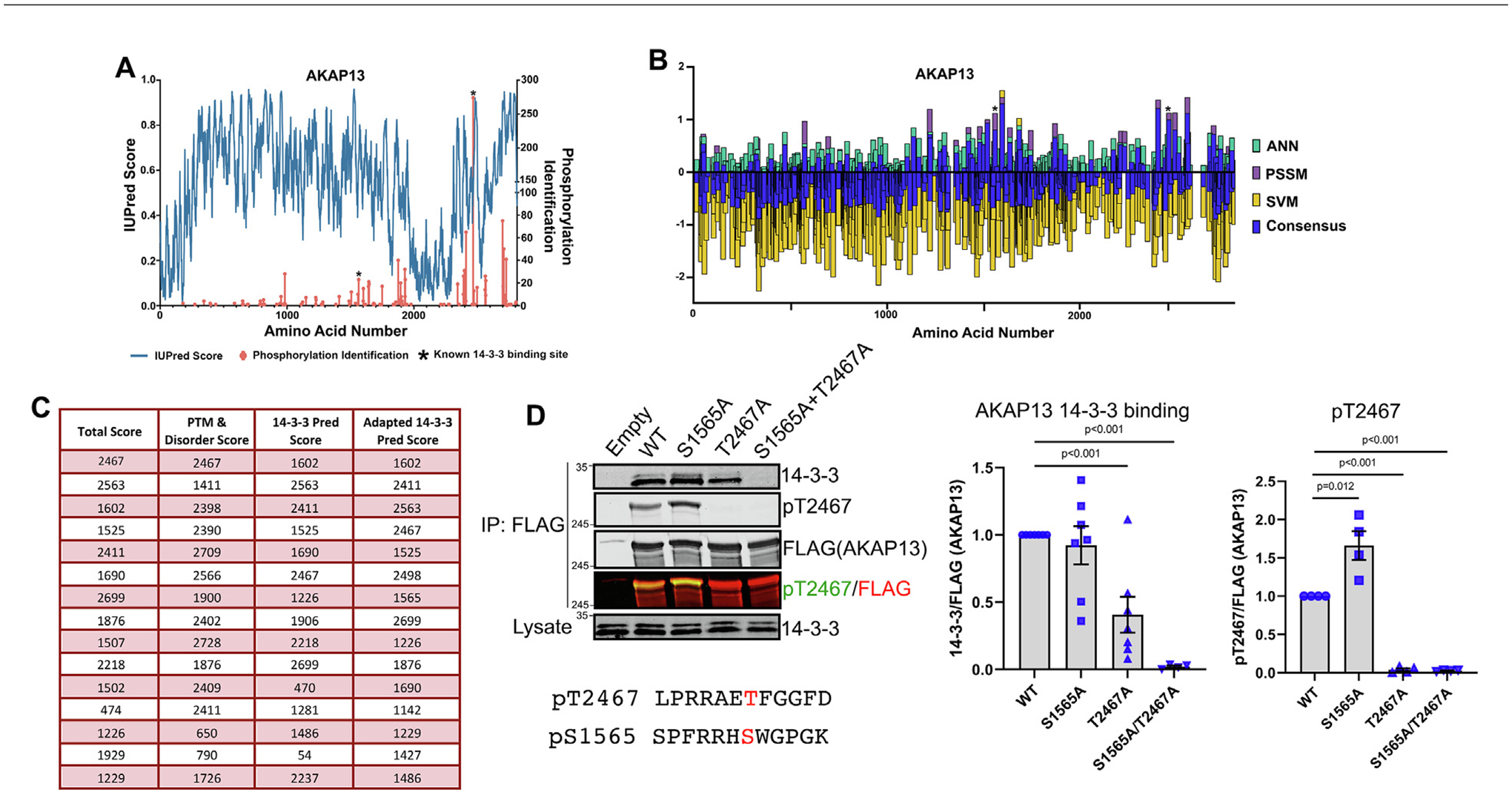
Identification of pT2467 as the dominant 14-3-3 docking site phosphorylation in AKAP13. (A) Disorder scores from IUPred and Ser/Thr phosphorylation identifications from phosphositeplus.org graphed against the amino acid numbers for AKAP13. (B) Summary bar graph of ANN, PSSM, SVM, and consensus scores from 14-3-3Pred graphed against the amino acid numbers of AKAP13. Asterisks in A-B indicate known 14-3-3 docking site. (C) Summary chart of the top 15 predicted docking sites for AKAP13 by 14-3-3-site-finder for each output described. (D) Indicated mutated constructs of AKAP13 were expressed in HEK293T cells followed by co-immunoprecipitation for 14-3-3 binding. Co-IP was immunoblotted for 14-3-3 and pT2467 signal. Quantification of multiple biological replicates of 14-3-3 binding to FLAG (AKAP13) and pT2467 on the right. Error bars indicate SEM for Students t-test.

A feature of the PTM-binding groove of 14-3-3 is its ability to accommodate two phosphorylations. The relative contribution of the two phosphorylations can vary, with some client proteins requiring both phosphorylations for 14-3-3 binding, and others only requiring one, leading to the idea of 14-3-3 as a molecular logic gate.^40,72^ For most experimentally identified 14-3-3 client proteins, only one docking site phosphorylation is known,^40,49^ yet it is likely that phosphorylations at a second site contribute to 14-3-3 binding on those proteins. In searching the list of 14-3-3 client proteins with only one identified docking site phosphorylation, we identified AKAP13 as an interesting candidate for identification of a second docking site phosphorylation. Diviani et al. used Scansite, an older iteration of 14-3-3 docking prediction software, to identify S1565 as required for 14-3-3 binding and found that 14-3-3 binding inhibits AKAP13 Rho-GEF activity.^73^ However, we found that AKAP13 has long regions of disorder with numerous phosphorylations, many of which are identified more frequently than S1565 in unbiased MS studies (Figure 8(A)). Furthermore, 14-3-3Pred does not rank S1565 highly (Figure 8(B) and (C)). Therefore, we questioned whether our approach could identify a second docking site phosphorylation in AKAP13. We excluded AKAP13 from our training set, retrained the tool, and predicted potential docking site phosphorylations across AKAP13. Our Total Score column (factoring in all inputs) identified phospho-residues at 2467, 2563, 1602, 1525, and 2411 as the top-predicted 14-3-3 docking sites. Interestingly, these sites are quite different than sites predicted by 14-3-3Pred alone, suggesting that the addition of protein disorder and PTM score significantly impact the rankings (Figure 8(C)). We then took the top site predicted by our tool, T2467, and mutated it in combination with S1565. Surprisingly, we found that a phospho-null mutation at S1565 (S1565A) alone had no significant effect on 14-3-3 binding to AKAP13, whereas mutation of T2467 (T2467A) alone abrogated 14-3-3 binding by more than 50%. Notably, the SAPH-ire approach^54,55,74^ from Figure 1 identified the T2467 site as the most likely functional PTM in the entire AKAP13 protein family. We also detected a robust phospho-signal using a custom phospho-antibody to pT2467, which increased upon mutation of S1565, suggesting an interplay between these two phosphorylations (Figure 8(D)). Combining the two mutations completely abrogated 14-3-3 binding (Figure 8(D)), suggesting that 14-3-3 is an “OR” logic gate for AKAP13 as both phosphorylations must be eliminated to completely abolish 14-3-3 binding.^40^ Together, these data highlight the strength of combining multiple protein features to identify novel 14-3-3 docking site phosphorylations.

## Discussion

A major challenge to PTM research is sifting through the reams of PTM proteomics data for biologically functional protein modifications, which often exist among numerous modifications that have seemingly little to no impact on protein function. In our view, the 14-3-3 interactome has the potential to accelerate this search, guiding us to phosphorylations that control core biological functions. However, in our experience, the most costly and time-consuming step has been identifying 14-3-3 docking site phosphorylations, often among numerous phosphorylation sites with similar sequences on a given protein. Our data suggest that combining the easily extractable protein features of intrinsic disorder and PTM observation frequency accelerates the identification of 14-3-3 docking site phosphorylations. We have now combined these features with consensus sequence prediction from 14-3-3Pred in a web app (https://rconnect.byu.edu/14-3-3-site-finder/) for the PTM and 14-3-3 research communities.

Factoring in disorder and PTM observation frequency worked particularly well for 14-3-3 docking sites that are poor matches to the consensus 14-3-3 docking sequences of RXXpS/TXP (mode I) or RXF/YXpSXP (mode II). Other groups have discussed in detail how these non-canonical 14-3-3 docking sequences have become relatively common,^35,51^ especially as the number of 14-3-3 interactors has increased with interactomics approaches. One example is TNK1, which has a 14-3-3 docking sequence that bears essentially no resemblance to the consensus sequence, yet the site is highly detected in unbiased MS studies and sits within a region of high predicted intrinsic disorder. Additional examples in Figure 5 include ESR1, CHEK1 and MAPK9. Interestingly, we noticed that a subset of these identified non-canonical sites either had relatively low PTM observation scores or were in regions of relatively low disorder. Despite this, the random forest model did a reasonably good job at identifying the sites, possibly due to some combination of these features recognized by the algorithm. One shortcoming to this approach is that some proteins, for various reasons (e.g., proteins that are in low abundance or hard to extract for MS), are more likely to have phosphorylations that are undetected in unbiased PTM-focused MS approaches, which eliminates an important variable for our approach. For now, until additional predictive protein features can be included, intrinsic disorder and consensus sequence matching may be the most reliably predictive variables in these instances.

Our results raise questions about why intrinsic disorder and PTM observation frequency are predictive for 14-3-3 docking site phosphorylations. We noted that for many 14-3-3 client proteins, there are long stretches of disorder on both sides of the phosphorylation. These disordered stretches likely help accommodate phosphorylated proteins into the rigid structure and phospho-binding grooves of 14-3-3, which are ~34 Å apart.^35,40,75^ Furthermore, phosphorylations with high MS observation frequency are likely abundant, high stoichiometry phosphorylations, which logically tend to be functionally important.^55,76^ Interestingly, our observation that 14-3-3 docking site phosphorylations are among the most observed phosphorylations on a given protein may refelect the role of 14-3-3 in protecting phosphorylations from phosphatase activity, which would naturally increase the MS observation frequency. This function of 14-3-3 has been demonsrated for a handful of client proteins, but is perhaps more common than we appreciated.^5,60–62^ Additionally, other protein structural or sequence features could be harnessed to improve 14-3-3 docking site predictions. For example, the structure of 14-3-3 bound to BRAF, as well as other 14-3-3 client protein co-structures, highlight contact sites that are distant from the 14-3-3 phospho-binding groove.^33,53,69,77,78^ However, fully realizing the predictive power of these additional features is limited by our incomplete structural understanding of many 14-3-3 client proteins, further complicated by the fact that many docking sites sit within disordered regions that are difficult to resolve by structural techniques.

In summary, this study provides a new tool to accelerate the discovery of 14-3-3 docking site phosphorylations, validated by the identification of sites that are poor matches to consensus sequence and the identification of a novel 14-3-3 docking site phosphorylation on AKAP13. Future work will focus on identifying additional features predictive for 14-3-3 binding. Understanding the full scope of what mediates 14-3-3 interactions will provide insight into how the specificity of 14-3-3 binding is controlled, and perhaps even the determinants of 14-3-3 isoform-specific interactions—a long standing question in 14-3-3 research. Ultimately, understanding how 14-3-3 regulates client proteins within its vast and still largely untapped interactome will continue to provide critical insight into fundamental biological processes and expand possibilities for promising new approaches, including molecular glues,^79–81^ to therapeutically modulate 14-3-3 interactions.

## Supplementary Material

Supplementary figure 1

Supplementary table/dataset 2

Supplementary table/dataset 1
