# Supplementary figures and images for: The Integration of Proteome-Wide PTM Data with Protein Structural and Sequence Features Identifies Phosphorylations that Mediate 14-3-3 Interactions
Source: J Mol Biol. Author manuscript; Available in PMC 2023 Apr 13. (PMC10099770; doi:10.1016/j.jmb.2022.167890)

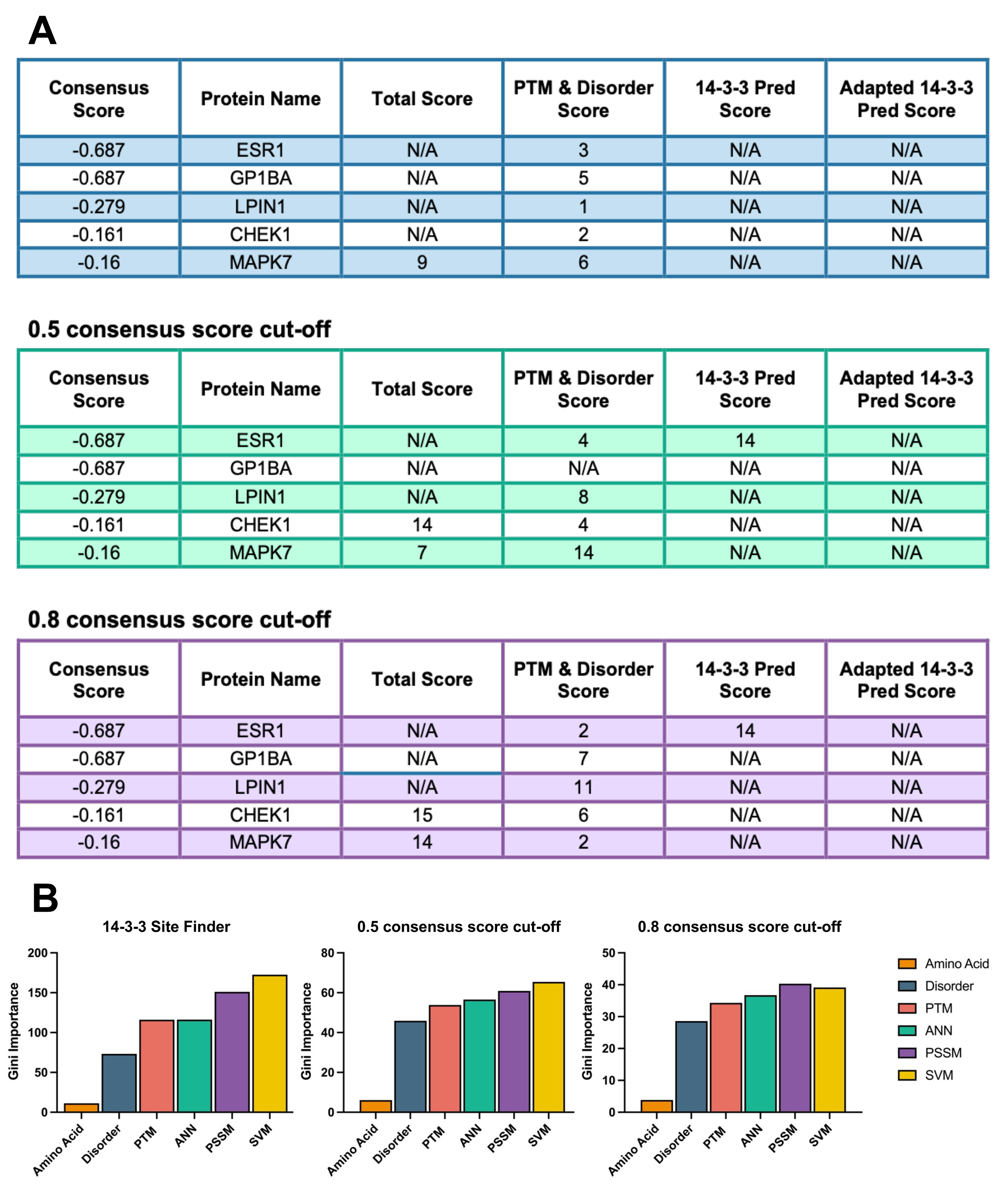

Supplement: Supplementary figure 1 [file NIHMS1881042-supplement-Supplementary_figure_1.jpg]
